# Need for improved detection of voluntary medical male circumcision adverse events in Mozambique: a mixed-methods assessment

**DOI:** 10.1186/s12913-019-4604-1

**Published:** 2019-11-21

**Authors:** Atanásio Brito, Abigail Korn, Leonel Monteiro, Florindo Mudender, Adelina Maiela, Jotamo Come, Scott Barnhart, Caryl Feldacker

**Affiliations:** 1 International Training and Education Center for Health (I-TECH), University of Washington, Av. Cahora Bassa #106, Maputo, Mozambique; 2 International Training and Education Center for Health (I-TECH), University of Washington, 908 Jefferson Street, 12th Floor, Seattle, WA 98104 USA; 3 Eduardo Mondlane University, School of Medicine, Av. Salvador Allende #702, Maputo, Mozambique; 4 National Male Circumcision Programme – Ministry of Health, Av. Eduardo Mondlane #1008, Maputo, Mozambique; 5 Department of Medicine, University of Washington, Seattle, WA USA; 6 Department of Global Health, University of Washington, Seattle, WA USA

**Keywords:** Voluntary medical male circumcision, Data quality, Adverse events, Mozambique

## Abstract

**Background:**

Adverse events (AE) resulting from voluntary medical male circumcision (VMMC) are commonly used to measure program quality. Mozambique’s VMMC program data reports a combined moderate and severe AE rate of 0.2% through passive surveillance. With active surveillance, similar programs report AE rates ranging from 1.0 to 17.0%. The objective of this activity was to assess potential underreporting of AEs via the passive surveillance system in Mozambique.

**Methods:**

This mixed-methods assessment randomly selected one third (16) of all 46 VMMC clinics through stratified sampling, based on volume. A retrospective record review was conducted including patient clinical files, stock records of Amoxicillin/Clavulanic Acid (the choice antibiotic for VMMC-related infections), and clinic-level AE rates from the national database. Records from the month of April 21 to May 20, 2017 were analyzed to identify both reported and potentially unreported AEs. In addition, external, expert clinicians observed post-operative visits (*n* = 167). Descriptive statistics were calculated, including difference between reported and identified AEs, an adjusted retrospective AE rate, and an observed prospective AE rate in each clinic.

**Results:**

A total of 5352 circumcisions were performed in the 16 clinics: 8 (0.15%) AEs were reported. Retrospective clinical record reviews identified 36 AEs (0.67%); AE severity or type was unknown. Using Amoxicillin/Clavulanic Acid dispensation as a proxy for VMMC-related infections, 39 additional AEs infections were identified, resulting in an adjusted AE rate of 1.4%, an 8.3 fold increase from the reported AE rate. Prospective, post-operative visit observations of 167 clients found 10 AEs (5.9%); infection was common and boys 10–14 years old represented 80% of AE clients.

**Conclusions:**

Evidence suggests underreporting of AEs in the Mozambican VMMC program. Quality improvement efforts should be implemented in all VMMC sites to improve AE identification, documentation and prevention efforts.

## Background

Three sub-Saharan African trials found that voluntary medical male circumcision (VMMC) reduces the risk of male acquisition of HIV from sexual intercourse with an HIV-infected woman by 60% [1–3]. To further reduce the spread of HIV in the region, subsequent VMMC support from the World Health Organization (WHO) and UNAIDS led to over 14.5 million MCs through 2016, and calls for another 25 million MCs among men ages 15–49 by 2020 [4–7]. Overall, VMMC is considered safe. Adverse events (AE) rates, a key indicator of VMMC service delivery quality, range from 0.5 to 7% in randomized control trials and active surveillance settings [1–3, 8–11]. Further meta-analysis results from a larger pool of VMMC programs recently found a pooled average AE proportion of 2.3% [12]. Together, clinical trial and subsequent VMMC program implementation led to a global expectation of approximately 2.0% AEs [13].

**Fig. 1:**
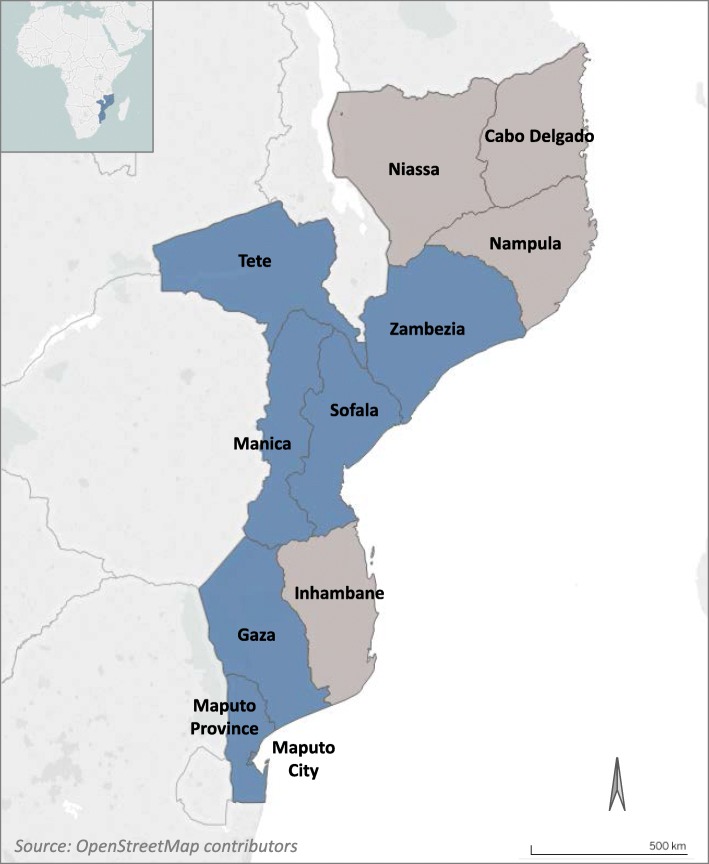
Map of the priority VMMC provinces (blue) of Mozambique (source: OpenStreetMap contributors)

In Mozambique, with an HIV prevalence of 13.2% among those sexually active [14], VMMC efforts are a critical component of comprehensive HIV prevention. In 2009, the Ministry of Health (MoH) of Mozambique adopted VMMC as a pillar of its HIV control strategy, with a focus on seven provinces: Zambézia, Tete, Manica, Sofala, Gaza, Maputo, and Maputo City (see Fig. 1). The main objective of the National Strategic Plan 2013–2017 was to circumcise 2 million men between 10 and 49 years old. By the end of 2017, 61% of this target was reached through services in 46 VMMC fixed units, 140 temporary units, and 16 mobile units [15]. According to the national strategy, three post-operative medical checks are scheduled after VMMC on Day 0: Day 2, Day 7 and Day 42. Key strategies for providing safe procedures and minimizing the risk of complications include developing and implementing quality assurance activities, ensuring provider competency, and monitoring continuous improvement of high quality, safe care [15].

In Mozambique, all VMMC clinics participate in an external quality assurance (EQA) program supported by the International Training and Education Center for Health (I-TECH)/Mozambique to help ensure adherence to MoH VMMC clinical guidelines. The EQA process consists of semi-annual visits to VMMC sites to measure standardized productivity and safety indicators. Each EQA is followed by routine follow-up visits aimed at program monitoring, adverse events, antibiotic stewardship, and mentoring tailored to site-specific improvement plans. The EQA process is implemented by integrated teams of VMMC clinicians and monitoring and evaluation specialists from I-TECH, MoH, and implementing partners.

Between 2013 and 2017, the average AE rate in Mozambique was reported at 0.26% [15]. In 2017, specifically, the MoH reported a total of 315,380 circumcisions and 537 AEs through passive surveillance (0.18%): 68 (13%) were severe (requiring surgical re-exploration or hospitalization), 415 (77%) were moderate (requiring intervention or medication, not categorized as severe), and 84 (16%) were mild (needing only reassurance or education (not reported)) [15]. AEs in Mozambique are much lower than the global average from field programs. There is concern that the routine passive surveillance system might be underreporting AEs. Therefore, I-TECH conducted this quality improvement activity with the objective of assessing the reliability of reported AE rates.

## Methods

### Design

A mixed-methods approach triangulated two retrospective data sources with prospective clinical observations in order to estimate potential underreporting of AEs. First, retrospective review of client records from April 21 to May 20, 2017 was conducted to confirm reported AEs as well as to identify potential unreported AEs. Second, antibiotic stock cards for the same time period were retrospectively reviewed for common AE treatments such as Amoxicillin/Clavulanic Acid (ACA), aiming to identify additional undocumented AEs. Third, prospective observations of post-operative visits were conducted by an expert clinician to ascertain site-based clinical skills and calculate an observed AE rate. Efforts were made to employ all three approaches at each of the 16 sites.

### Setting and sampling

The sampling frame of all 46 VMMC clinics in Mozambique were stratified based on high or low volume. High-volume clinics were defined as those that performed a monthly average of 250 procedures or more through February 2017. One third of VMMC clinics were randomly selected in proportion to high- and low-volume clinics within each stratum using the random number formula in Excel®. Clinics are only identified by province to reduce site identification.

### Study procedure

Teams included three clinicians (either surgical technicians or medical doctors trained for VMMC), as well as three monitoring and evaluation technicians (public health technicians, epidemiologists, or statisticians). First, the teams reviewed all VMMC records (*ficha de utente de CMMV)* for the one-month period, April 21 to May 20, 2017, to document evidence of reported or suspected (unreported) AEs. Reviews also included data gathered from provider comments and treatment documentation pertinent to adverse events (ACA prescription, use of iodine ointment, application of stitches, etc.). Second, in nine clinics with available stock cards, teams reviewed them for use of ACA, the treatment promoted by the MoH for moderate to severe infections. According to MoH guidelines, each client diagnosed with an infection (generally mild and moderate) is prescribed 21 ACA tablets over the course of a 7-day treatment period [16]. Teams compared the dispensation of ACA to the number of infections reported during the review period. Third, experienced VMMC clinicians from both implementing partners and MoH observed post-operative client visits, either routine or spontaneous. Experienced VMMC clinicians observed site VMMC teams, providing input and mentorship if the site provider requested support or incorrectly assessed the type or severity of any AE. Observed clients with AEs were identified, classified, and reported according to MoH guidelines in tandem with site teams. Data collected from post-operative observations included routine data (AE by type and severity [16]) and the team’s qualitative assessment of provider skill to identify, classify severity, and prescribe adequate AE treatment.

### Data analysis

Descriptive statistics were calculated using SPSS Statistics 21 (IBM Corp, USA). The difference (count) between reported and identified AEs over the 1 month retrospective review period was calculated. The suspected number of VMMC AEs due to infections was estimated using the quantity of ACA dispensed. An *adjusted, retrospective AE rate* during the period of review was estimated by adding the number of suspected VMMC-related infections from ACA dispensing records to the number of AEs (both reported and newly identified) at each site, divided by MCs at each site over the period. An *observed AE rate* was calculated by dividing the number of observed clients with a moderate or severe AE by the total number of observed clients receiving follow-up VMMC care.

## Results

### Evaluation of client clinical records

A total of 5352 male circumcisions were performed in the 16 selected clinics during the 1 month period of review, and 8 (0.15%) AEs were reported to the MoH (Additional file 1): 4 hematomas, 2 infections, 1 delayed wound healing, and 1 suture dehiscence. Clinical record review identified 28 additional AEs (not previously reported), for a total of 36 AEs. Lack of detailed documentation on either wound condition or treatment precluded accurate classification of type or severity of new AEs. In eight clinics (50%), no AEs were found through clinical records review or MoH reports.

### Stock card review

According to the total quantity of ACA tablets dispensed at nine clinics with available stock cards, 39 clients were suspected of VMMC-related infections. There were suspected AEs found at all clinics where ACA review was conducted; however, no infections were reported in the nine clinics. Of the seven clinics that did not have stock cards available for review; one clinic reported two infections (Additional file 1). Inclusion of these additional unreported AE resulted in an estimated average AE rate of 1.4%.

Inclusion of presumed AEs from ACA dispensing in addition to AEs identified from clinical records review, increased AE rates at most clinics (Table 1). Three clinics increased their AE rates to over 5.0%. Two clinics (#3 and #4) conducted only 15% of all VMMC procedures, but combined to generate 41% of all infections suggested by ACA prescriptions. Of all 39 suspected infections from ACA, 9 (23%) were from one site where no AEs were reported to MoH nor found through clinical record review.

**Table 1:** Reported and adjusted AE rates from retrospective data: April 21–May 20, 2017

Site	Reported AE Rate (%)	Adjusted AE Rate (%)
1	1	1.3
2	0.29	0.29
3	0.27	5.1
4	0.24	5.1
5	0.14	0.14
6	0	5.3
7	0	2.6
8	0	2
9	0	1.6
10	0	0.83
11	0	0.6
12	0	0.49
13	0	0
14	0	0
15	0	0
16	0	0
Total/avg.	0.15	1.4

### Prospective observation of client post-operative visits

A total of 167 clients were observed during follow-up visits; ten (6%) AEs were found, three severe and seven moderate. Among the seven moderate AEs, there was one excessive bleeding on Day 1, one bleeding on Day 13, three infections on Day 7, and two infections on Day 11. Of the three severe AEs, one hematoma was reported on Day 1, one excessive skin removal (likely intra-operatively) found on Day 8, and one suture dehiscence on Day 15. Of all post-operative observations, 79 (47.0%) were among boys ages 10–14 years. These young clients comprised 8 (80.0%) of the total observed severe and moderate AEs. Of 85 clients observed for the Day 2 visit, 31 (36.5%) of clients returned on their scheduled date; 29 (34.1%) returned on Day 1, and the remaining 25 clients (29.4%) returned on Day 3 or 4. Among the 82 clients observed for the Day 7 follow up visit, 61 (74%) returned on Day 7, three (3.7%) returned early for scheduling reasons, and the remaining 14 (17%) clients returned up to 8 days after their scheduled appointment. All AEs identified by the team through post-operative observations were independently identified and classified correctly by the site providers.

## Discussion

Prompt and correct identification of AEs is important to ensure quality care for clients and to implement appropriate preventive measures across the program. This project assessed the reliability of routine AE reporting using both retrospective and prospective methods, suggesting that AEs from VMMC clients are likely underreported. Moderate and severe AEs from both retrospective client record review and ACA dispensing were 8.3-fold (0.15 vs 1.4%) higher than those reported to MoH from passive surveillance. A 38-fold increase was found when comparing the average reported AE rate to that observed during the prospective post-operative wound care observations (0.15% vs 5.9%). Triangulation of data used for this activity lends strength to the findings and suggests that underreporting of AEs is evident in these clinics. It should be noted, however, these increased rates, in absolute terms, were modest and within the range of other reported rates and acceptable standards. These results lead to several discussion points and suggested actions.

First, the increase in AE rates found in this exercise are not evenly distributed. Several clinics account for a disproportionate number of AEs found both retrospectively and prospectively. All three severe AEs found during active surveillance and 23% of all suspected infections from ACA dispensing were observed at one high volume site. Another high-volume site accounted for four of seven moderate AEs, mostly infections. Two other high volume sites contributed nearly 53% of all suspected and reported AEs, despite conducting only 15% of all VMMC procedures. Although high volume sites conduct more MCs and, therefore, should expect to have more AEs, an AE rate above 2.0% is higher than the commonly accepted global standard [18] and suggests review for possible quality improvement intervention. Follow-up should help ensure that safety is emphasized as productivity is maintained. Furthermore, the newly identified AEs from retrospective data suggest problems in the routine monitoring and reporting systems.

Provider training and/or behavior likely contributes to underreporting of AEs. First, MoH VMMC program standards require reporting of only moderate or severe complications. This may influence the misclassification of AEs towards mild, reducing the need for recordkeeping or reporting. Furthermore, providers may not record information according to MoH standards. The study team noted that recorded information in the client’s form (“*ficha de utente CMMV*”) varied widely in terms of completeness and detail, revealing a gap in data quality. Providers also appear to use the client form to suggest patient follow-up steps for other providers, internally, rather than reporting AEs. A provider refresher training and close supervision may help ameliorate potential provider-related issues in AE identification and documentation.

Furthermore, antibiotic stewardship appears poor in many of the clinics where stock cards were reviewed. ACA is the most widely used antibacterial agent in Mozambique’s VMMC program and should be prescribed when there is clear evidence of moderate infection [18]. Pharmacy records have been found to be a reliable estimate of ART coverage in Malawi [19] and of nosocomial infections in the United States [20]. Guided by previous research, this project used antibiotic dispensing as a proxy indicator for detecting VMMC-related infections. Although ACA, or antibiotic dispensing in general, is not a foolproof indicator of infection [10], identification of 39 unreported VMMC-related infections is cause for concern. No misuse of ACA (e.g. for prophylactic use), was reported by the EQA performed in Mozambique by I-TECH, MoH and implementing partners, suggesting that these findings reflect true underreporting of infections. Additional training for providers in reporting of infections and routine review of ACA dispensing could improve AE detection due to infection.

Lastly, potential underreporting of AEs is not unique to field settings in Mozambique. Another large-scale VMMC program in Zimbabwe reported similarly low AE rates [17], while studies comparing passive to active surveillance in field settings in Kenya confirm the likelihood of underreporting of AE cases through routine systems [18, 21, 22]. Field studies in other sub-Saharan African settings that report low AE rates (< 2%) also found sub-optimal retention in post-procedure follow-up [23–27], especially after Day 2, suggesting that some AEs may not be identified, in part as clients may seek care outside of routine VMMC settings. Indeed, experiences in Kenya [8] and Botswana [11] with high retention found more AEs, with AE rates of 5.1 and 6.7%, respectively. Although Mozambique national VMMC data from 2013 to 2017 report that Day 2 follow-up rates increased from 25% (2013) to 80% (2017) and that Day 7 follow-up increased from 15% (2013) to 64% (2017) [15], there remains a large proportion of men who do not return to routine VMMC settings, some of whom may have undetected - and unreported - AEs.

### Limitations

This study has several limitations. Stock cards were not readily available in 7 clinics; therefore, only nine clinics contributed data on ACA dispensing. No other antibiotic dispensing records were reviewed; therefore, AEs treated with other drugs such as Amoxicillin are unknown. Also, due to lack of detailed descriptions in clinical records, clinical evaluators could not determine AE severity nor type from retrospective review; thus the additional AEs may include some mild along with moderate and severe. In addition, sites have little control over a client’s desire to access and practice good wound hygiene, limiting the linkages between site-based capacity and individual action. Lastly, as with most quality assurance activities in resource constrained field settings, the sample size is small. Therefore, we are unable to generalize with certainty beyond the included clinics or compare these findings quantitatively with other studies on AEs with Mozambique or the region. Despite these limitations, the methods and findings from this study strongly suggest that AEs are underreported, a finding that is relevant to other large VMMC programs in Mozambique and in the region.

## Conclusions

Retrospective review of patient files and antibiotic stock records found an increase of 8.3 to 38-fold between previously-reported and newly-identified AEs. Prospective observations found an AE rate of 6%, as compared to a previously reported 0.15% AE rate, further suggesting underreporting of moderate and severe VMMC AEs. To address these discrepancies and improve AE reporting in the future, several next steps are suggested. First, there is a need to increase site-level supervision and encourage VMMC providers to report all AEs observed regardless of severity. Providers should be informed that reporting AEs is a sign of high-quality VMMC program implementation and that reporting will be viewed positively. Second, raising awareness about the importance of correctly and completely reporting complications on routine reporting forms is needed. MoH should lead this reporting form training effort with both providers and support staff including data clerks. Third, dissemination of standardized reporting procedures is needed, including clarification of provider reporting responsibilities in cases where multiple providers or sites are involved in a client’s care. This determination of duties and lines of authority would further strengthen supervision efforts. Further study is warranted to determine root causes for AE underreporting, leading to improved AE identification and documentation.

## Supplementary information

**Additional file 1.** Map of the priority VMMC provinces of Mozambique. Mozambique’s priority provinces for VMMC programming are shown in blue.

## Data Availability

The data that support the findings of this study are available from the Mozambique Ministry of Health but restrictions apply to the availability of these data, which were used under I-TECH’s authorization for the current study, and so are not publicly available.

## Abbreviations

ACA: Amoxicillin/Clavulanic Acid
AE: Adverse Event
EQA: External Quality Assurance
HIV: Human Immunodeficiency Virus
I-TECH: International Training and Education Center for Health
MC: Male Circumcision
MoH: Ministry of Health
UNAIDS: Joint United Nations Programme on HIV/AIDS
VMMC: Voluntary Medical Male Circumcision
WHO: World Health Organization

## References

[1] Auvert B, Taljaard D, Lagarde E, Sobngwi-Tambekou J, Sitta R, Puren A (2005). Randomized, controlled intervention trial of male circumcision for reduction of HIV infection risk: the ANRS 1265 trial. PLoS Med.

[2] Bailey RC, Moses S, Parker CB, Agot K, Maclean I, Krieger JN (2007). Male circumcision for HIV prevention in young men in Kisumu, Kenya: a randomised controlled trial. Lancet.

[3] Gray RH, Kigozi G, Serwadda D, Makumbi F, Watya S, Nalugoda F (2007). Male circumcision for HIV prevention in men in Rakai, Uganda: a randomised trial. Lancet.

[4] World Health Organization (2015). WHO Progress Brief: Voluntary Medical Male Circumcision for HIV Prevention In 14 Priority Countries in East And Southern Africa.

[5] World Health Organization. Voluntary Medical Male Circumcision For HIV Prevention In 14 Priority Countries In East And Southern Africa Geneva: WHO; 2016. Available from: http://apps.who.int/iris/bitstream/10665/246174/1/WHO-HIV-2016.14-eng.pdf. Cited 2017 March 28

[6] World Health Organization (2017). Voluntary Medical Male Circumcision For HIV Prevention In 14 Priority Countries In Eastern And Southern Africa.

[7] World Health Organization (2016). A framework for voluntary medical male circumcision: effective HIV prevention and a gateway to improved adolescent boys’ & men’s health in Eastern and Southern Africa by 2021.

[8] Reed JB, Grund J, Liu Y, Mwandi Z, Howard AA, McNairy ML (2015). Implementation and operational research: evaluation of loss-to-follow-up and postoperative adverse events in a voluntary medical male circumcision Programme in Nyanza Province, Kenya. J Acquir Immune Defic Syndr.

[9] Frajzyngier V, Odingo G, Barone M, Perchal P, Pavin M (2014). Safety of adult medical male circumcision performed by non-physician clinicians in Kenya: a prospective cohort study. Glob Health Sci Pract.

[10] Kohler PK, Namate D, Barnhart S, Chimbwandira F, Tippet-Barr BA, Perdue T (2016). Classification and rates of adverse events in a Malawi male circumcision programme: impact of quality improvement training. BMC Health Serv Res.

[11] Wirth KE, Semo B-W, Spees LP, Ntsuape C, Barnhart S, Ledikwe JH (2017). A prospective cohort study of safety and patient satisfaction of voluntary medical male circumcision in Botswana. PloS One.

[12] Ford N, Chu K, Mills EJ (2012). Safety of task-shifting for male medical circumcision: a systematic review and meta-analysis. Aids.

[13] World Health Organization (2013). WHO Technical Advisory Group on Innovations in Male Circumcision: Evaluation of two adult devices: Meeting report.

[14] Reed JC (2018). Preliminary Report on HIV Indicators. IMASIDA - Malaria HA, and Immunization Indicator Survey in Mozambique 2015.

[15] Ministry of Health Mozambique (2018). Relatório Anual das Actividades Relacionadas a Circuncisão Médica (Annual Report on Activities Related to Medical Circumcision).

[16] Mozambique MH (2017). Manual de Referencia Para Formacao Tecnica Sobre Circuncisao Medica.

[17] President’s Emergency Plan for AIDS Relief (2017). PEPFAR’s Best Practices for Voluntary Medical Male Circumcision Site Operations: A service guide for site operations. Managing, Monitoring, And Reporting VMMC Adverse Events.

[18] Population Services International CoSoE, Central and Southern Africa (COSECSA), U.S. Centers for Disease Control and Prevention (2016). Adverse Event Action Guide for Voluntary Medical Male Circumcision by Surgery or Device.

[19] Tweya H, Feldacker C, Ben-Smith A, Harries AD, Komatsu R, Jahn A (2012). Simplifying ART cohort monitoring: Can pharmacy stocks provide accurate estimates of patients retained on antiretroviral therapy in Malawi?. BMC Health Serv Res.

[20] Hirschhorn LR, Currier JS, Platt R (1993). Electronic surveillance of antibiotic exposure and coded discharge diagnoses as indicators of postoperative infection and other quality assurance measures. Infect Control Hosp Epidemiol.

[21] Bochner A, Feldacker C, Makunike B, Holec M, Murenje V, Stepaniak A (2017). Adverse event profile of a mature voluntary medical male circumcision progra mme performing PrePex and surgical procedures in Zimbabwe. J Int AIDS Soc.

[22] Herman-Roloff A, Bailey RC, Agot K (2012). Factors associated with the safety of voluntary medical male circumcision in Nyanza province, Kenya. Bull World Health Organ.

[23] Phili R, Abdool-Karim Q, Ngesa O (2014). Low adverse event rates following voluntary medical male circumcision in a high HIV disease burden public sector prevention programme in South Africa. J Int AIDS Soc.

[24] Wynn A, Bristow CC, Ross D, Schenker I, Klausner JD (2015). A programme evaluation report of a rapid scale-up of a high-volume medical male circumcision site, KwaZulu-Natal, South Africa, 2010–2013. BMC Health Serv Res.

[25] Lissouba P, Taljaard D, Rech D, Doyle S, Shabangu D, Nhlapo C (2010). A model for the roll-out of comprehensive adult male circumcision services in African low-income settings of high HIV incidence: the ANRS 12126 Bophelo Pele project. PLoS Med.

[26] Mahler HR, Kileo B, Curran K, Plotkin M, Adamu T, Hellar A (2011). Voluntary medical male circumcision: matching demand and supply with quality and efficiency in a high-volume campaign in Iringa region, Tanzania. PLoS Med.

[27] Montague C, Ngcobo N, Mahlase G, Frohlich J, Pillay C, Yende-Zuma N (2014). Implementation of adolescent-friendly voluntary medical male circumcision using a school based recruitment programme in rural KwaZulu-Natal, South Africa. PloS One.

