# Supplementary material for: Need for improved detection of voluntary medical male circumcision adverse events in Mozambique: a mixed-methods assessment
Source: BMC Health Serv Res. 2019 Nov 21;19:855. doi: 10.1186/s12913-019-4604-1 (PMC6868762; doi:10.1186/s12913-019-4604-1)
Supplement: Supplementary file 1 — Additional file 1. Map of the priority VMMC provinces of Mozambique. Mozambique’s priority provinces for VMMC programming are shown in blue. [file 12913_2019_4604_MOESM1_ESM.docx]

**Additional file 1. Reported and unreported AEs from retrospective data: April 21 - May 20, 2017**

| Site | Total MCs | Reported AE Rate | Reported AEs | Previously unidentified AEs | | | | Total AEs | New AE rate^†^ | |
| --- | --- | --- | --- | --- | --- | --- | --- | --- | --- | --- |
|  | (#) | (%) | (#)  (a) | Chart review  (#)  (b) | ACA stock card  (#)  (c) | | | (#)  (a+b+c) | (%) | |
| 1 | 301 | 1 | 3 | 0 | 1 | | | 4 | 1.3 | |
| 2 | 682 | 0.29 | 2 | 0 | * | | | 2 | 0.29 | |
| 3 | 374 | 0.27 | 1 | 9 | 9 | | | 19 | 5.1 | |
| 4 | 409 | 0.24 | 1 | 13 | 7 | | | 21 | 5.1 | |
| 5 | 694 | 0.14 | 1 | 0 | * | | | 1 | 0.14 | |
| 6 | 94 | 0 | 0 | 0 | 5 | | | 5 | 5.3 | |
| 7 | 156 | 0 | 0 | 1 | 3 | | | 4 | 2.6 | |
| 8 | 150 | 0 | 0 | 1 | 2 | | | 3 | 2 | |
| 9 | 549 | 0 | 0 | 0 | 9 | | | 9 | 1.6 | |
| 10 | 480 | 0 | 0 | 4 | * | | | 4 | 0.83 | |
| 11 | 164 | 0 | 0 | 0 | 1 | | | 1 | 0.6 | |
| 12 | 408 | 0 | 0 | 0 | 2 | | | 2 | 0.49 | |
| 13 | 507 | 0 | 0 | 0 | * | | | 0 | 0 | |
| 14 | 220 | 0 | 0 | 0 | * | | | 0 | 0 | |
| 15 | 94 | 0 | 0 | 0 | * | | | 0 | 0 | |
| 16 | 70 | 0 | 0 | 0 | * | | | 0 | 0 | |
| Total/avg. | 5,352 | 0.15 | 8 | 28 | | 39 | 75 | | 1.4 |  |

*No ACA stock card data available

**^†^** (a+b+c)/MCs
